# The Features of Immune Checkpoint Gene Regulation by microRNA in Cancer

**DOI:** 10.3390/ijms23169324

**Published:** 2022-08-18

**Authors:** Fatimat Kipkeeva, Tatyana Muzaffarova, Alexandra Korotaeva, Danzan Mansorunov, Pavel Apanovich, Maxim Nikulin, Olga Malikhova, Ivan Stilidi, Alexander Karpukhin

**Affiliations:** 1 Research Centre for Medical Genetics, 1 Moskvorechye St., 115522 Moscow, Russia; 2 N.N. Blokhin National Medical Research Center of Oncology, Ministry of Health of Russia, 24 Kashirskoe Shosse, 115478 Moscow, Russia

**Keywords:** microRNA, immune checkpoint, immunotherapy

## Abstract

Currently, the search for new promising tools of immunotherapy continues. In this regard, microRNAs (miRNAs) that influence immune checkpoint (IC) gene expression in tumor and T-cells and may be important regulators of immune cells are considered. MiRNAs regulate gene expression by blocking mRNA translation. An important feature of miRNA is its ability to affect the expression of several genes simultaneously, which corresponds to the trend toward the use of combination therapy. The article provides a list of miRNAs acting simultaneously on several ICs and miRNAs that, in addition to IC, can regulate the expression of targeted therapy genes. There is dependence of miRNA interactions with IC genes on the type of cancer. The analysis of the accumulated data demonstrates that only about 14% (95% CI: 9.8–20.1%) of the studied miRNAs regulate the expression of specific IC in more than one type of cancer. That is, there is tumor specificity in the miRNA action on ICs. A number of miRNAs demonstrated high efficiency in vitro and in vivo. This indicates the potential of miRNAs as promising agents for cancer immunotherapy. Additional studies of the miRNA–gene interaction features and the search for an optimal miRNA mimic structure are necessary.

## 1. Introduction

Immunotherapy is an innovative method of cancer treatment. As a result of experiments and clinical trials, it has been found that immunotherapy can increase progression-free survival and overall survival. However, this method of treatment is effective in a limited number of patients, and in addition, it can cause severe adverse reactions due to hyperreactivity of the immune system [1]. In this regard, research is underway to develop new therapeutic approaches based on targeting immune checkpoints (ICs). 

ICs are regulators of the immune system, which are divided into stimulating and inhibitory molecules. Stimulating and inhibitory ICs regulate T-cell activation. Tumor cells have the ability to generate ligands that can bind to co-inhibitory receptor molecules. This interaction suppresses the antitumor immune response, allowing the tumor to “escape” from the immune system. As an opportunity to solve this problem, the blockade of ICs is used [2].

IC inhibitors are monoclonal antibodies that affect a specific target [3]. In order to increase the effectiveness of immunotherapy, the FDA approved a number of regimens, including a combination of two IC inhibitors, a combination of IC inhibitors and targeted therapy drugs, as well as antitumor bispecific antibodies [4,5]. It has been shown that in combination therapy regimens, patients experienced a higher response rate compared to monotherapy [6].

In addition, the search for a more promising immunotherapy approach is currently ongoing. In this regard, microRNAs (miRNAs) are considered. MiRNAs participate in tumor-cell signaling pathways and regulate many processes, including the antitumor immune response.

MiRNAs are small non-coding RNAs that carry out post-transcriptional regulation of gene expression. According to recent studies, miRNAs influence IC gene expression and are important regulators in both T-cells and tumor cells [7]. MiRNAs regulate gene expression by binding to the 3’-UTR of their mRNA [8,9,10]. MiRNAs can also affect IC expression indirectly, through molecules of different signaling pathways, such as PTEN, IFR-1, and others [11]. It is also important that one miRNA can affect several genes [7,12].

This article presents a review of miRNAs that interact with IC genes, analyzes their regulating IC expression in tumors of various types of cancer, and identifies miRNAs that act on several IC genes simultaneously. Due to these properties, miRNA-based therapy may become an alternative to the combination of targeted drugs in the future. The effect of miRNAs on the same IC gene in tumors of several types was analyzed. Thus, the tumor specificity of the miRNA–IC gene interaction was assessed. In addition, miRNAs are considered that are capable of simultaneously regulating the expression of targeted therapy genes along with IC genes. These issues have not been previously analyzed in existing reviews of miRNAs as IC regulators [13,14,15,16,17].

## 2. Immune Checkpoints

### 2.1. PD-1, PD-L1 and CTLA-4

Programmed cell death protein 1 (PD-1) and programmed death-ligand 1 (PD-L1) are the most studied members of the ICs. PD-1 and CTLA-4 receptors are co-inhibitory molecules. They are normally expressed on activated T-lymphocytes. The PD-1 receptor has PD-L1 and PD-L2 ligands, and the CTLA-4 receptor has CD80/CD86 ligands that can be expressed on tumor cells in various types of cancer. The interaction of PD-1 and CTLA-4 receptors with their ligands leads to suppression of the cytolytic activity of T-lymphocytes, which blocks antitumor immunity. The use of PD-1 and CTLA-4 checkpoint inhibitors increased patient survival compared with traditional chemotherapy in a number of studies, including studies on kidney cancer, melanoma, head and neck squamous cell cancer (HNSCC) and non-small cell lung cancer [18].

Based on preclinical and clinical trials, the FDA approved the use of PD-1 inhibitor-nivolumab in combination with CTLA-4 inhibitor (ipilimumab) for the treatment of several types of metastatic cancer [19].

### 2.2. Gal-9/Tim-3

One of the relatively new targets of immunotherapy is the T-cell immunoglobulin and mucin domain-3 (Tim-3)/Galectin-9 (Gal-9) pathway. Tim-3 is present on activated effector T-cells. It is an immunosuppressive receptor, causing exhaustion of T-cells [20]. A high level of Tim-3 expression in kidney cancer is associated with an unfavorable prognosis [21,22]. One of the ligands of Tim-3 is Gal-9, which belongs to the galectin family. It is reported that this family of proteins regulates tumor proliferation, migration, and metastasis [23].

Kidney, brain, colon, blood, liver, prostate, lung, and skin cancer cell lines were found to express detectable amounts of Tim-3 and Gal-9 proteins. It is assumed that the Tim-3/Gal-9 pathway is involved in the prevention of antitumor immunity [24]. However, the information regarding Gal-9 is contradictory. According to a number of authors, Gal-9 overexpression correlates with a poor prognosis in many types of cancer [25,26,27,28,29]. On the other hand, increased expression of Gal-9 has been shown to be associated with a favorable prognosis in solid tumors [30].

Currently, Gal-9 is considered as a target of immunotherapy, anti-Gal-9 antibodies have been developed. In the co-cultivation of T-cells and tumor cells, these antibodies contributed significantly to T-cell-mediated destruction of tumor cells [31].

In addition, considering Gal-9 as a target for immunotherapy, it should be taken into account that Gal-9 also interacts with stimulatory receptors, such as the 4-1BB-co-stimulating receptor of the tumor necrosis factor receptor superfamily (TNFRSF). Another member of the TNFRSF—GITR—may also be relevant for the results of Gal-9 inhibition [25]. 

### 2.3. VISTA

V-domain Ig-containing suppressor of T-cell activation (VISTA) is an immunosuppressive receptor. It is considered as a potential target of immunotherapy. VISTA expression level is significantly increased in clear cell renal cell carcinoma (ccRCC). In animal models of kidney cancer, VISTA blockade significantly suppressed tumor growth [32].

In addition, it was shown that the combination of a VISTA inhibitor with an TLR agonist led to the development of antitumor immunity associated with T-cells [33]. It is also reported that simultaneous blockade of CTLA-4 and VISTA can enhance the antitumor immune response in HNSCC [34].

### 2.4. BTLA

The B- and T-lymphocyte attenuator (BTLA) is expressed by most lymphocytes. BTLA belongs to the CD28 superfamily and is similar in structure and function to PD-1 and CTLA-4 [35]. Increased BTLA expression is associated with an unfavorable prognosis [36]. The BTLA ligand is HVEM (TNFRSF14), a mediator of herpes virus penetration, a membrane protein that belongs to the superfamily of tumor necrosis factor receptors. The BTLA/HVEM axis is one of the most important ICs [37].

### 2.5. B7-H3

B7-H3 (CD276) is a member of the B7 family. It functions as a co-stimulating and as a co-inhibiting immunoregulatory protein [38]. B7-H3 is not only a regulator of the antitumor immune response, but is also involved in angiogenesis and metastasis. Co-expression of B7-H3 and tyrosine kinase receptor of angiopoietin Tie-2 was detected in RCC. It was shown that overexpression of B7-H3 and Tie-2 in the vascular endothelium of RCC was associated with the density of tumor microvessels and disease progression [39]. Additionally, it was found that B7-H3 knockdown eliminated the pro-metastatic effect of fibronectin and significantly suppressed the metastasis of ccRCC cells [40].

Clinical trials have evaluated the effectiveness of B7-H3 inhibitors in the treatment of solid tumors, both as monotherapy and as part of combined therapy [41]. In particular, a bispecific anti-B7-H3/PD-1 fusion protein has been developed. It interacts simultaneously with the tumor-associated marker B7-H3 and the immunosuppressive signaling pathway PD-1/PD-L1, and also enhances antibody-dependent cellular cytotoxicity. Treatment with anti-B7-H3/PD-1 fusion protein leads to effective suppression of tumor growth in animal models of several types of cancer [42]. A bispecific antibody targeting B7-H3 and 4-1BB (BsAb; B7-H3 × 4-1BB) has also been developed, which is a B7-H3 inhibitor and a 4-1BB agonist. It has been shown that BsAb; B7-H3 × 4-1BB, as well as its combination with anti-PD-1 therapy, inhibits tumor growth in animal models of several types of cancer [43].

### 2.6. ICOS/ICOSL

Inducible co-stimulator (ICOS), a molecule that also belongs to the CD28/CTLA-4/B7 immunoglobulin superfamily. ICOS and its ligand (ICOSL) are involved in various aspects of the T-cell response. The ICOS/ICOSL pathway has been shown to play a significant role in anti-CTLA-4 therapy [44]. ICOS/ICOSL is considered as a target for kidney cancer in combination with anti-PD-L1 therapy (NCT03829501) [45].

### 2.7. LAG3

Lymphocyte-associated gene 3 (LAG3) is a transmembrane protein that is expressed on immune cells. LAG3 refers to immunosuppressive IC molecules. LAG3 overexpression is associated with overall survival, as is the overexpression of PD-1 and CTLA-4. Currently, bispecific antibody immunotherapy trials that aim to simultaneously inhibit LAG3 and PD-1/PD-L1/CTLA-4 are being conducted [46]. According to Zelba et al., simultaneous blocking of PD-1 and LAG3 is a promising strategy in the treatment of kidney cancer [47]. Fibrinogen-like protein 1 (FGL1) participates in inactivation of T-cells and is considered as the main ligand of LAG3 [48].

## 3. Simultaneous Inhibition of ICs and Other Targets

### 3.1. ICs and VEGF

Combined treatment regimens, including IC inhibitors and anti-VEGF therapy, have shown significant efficacy in patients with various types of cancer [49,50,51]. To date, antiangiogenic therapy in combination with anti-PD-1/PD-L1 (Pembrolizumab plus Axitinib, Nivolumab plus Cabozantinib and Pembrolizumab plus Lenvatinib) is recommended by the NCCN and EAU along with anti-PD-1/anti-CTLA-4 treatment (Ipilimumab plus Nivolumab as first-line therapy for metastatic RCC [52]).

### 3.2. ICs and c-Met

c-Met is a receptor tyrosine kinase that is involved in normal cell development and motility. Aberrant activation of c-Met can lead to tumor growth and metastasis [53]. The combination of ICI with cabozatinib, which inhibits c-Met in addition to VEGFR, is now recognized as the new standard of care for metastatic RCC [54]. There is also a study evaluating the efficacy of a combination of c-Met inhibitor and anti-PD-1 therapy in locally advanced or metastatic hepatocellular carcinoma and RCC (NCT03655613; NCT02795429).

### 3.3. ICs and HIF

Co-suppression of IC and Hypoxia-inducible factor (HIF) is also being considered as a therapeutic strategy. HIF activates downstream effectors, including vascular endothelial growth factor (VEGF), platelet growth factor (PDGF) and carbonic anhydrase IX (CA IX), which are involved in cell proliferation, angiogenesis, and erythropoiesis [55]. HIF-2α is considered as a target for therapy in RCC. The combination of a HIF-2α inhibitor (belzutifan), an anti-VEGF therapy (lenvatinib), and PD-1 and CTLA-4 inhibitors is currently being investigated in patients with RCC (NCT04736706).

### 3.4. ICs and PI3K

The efficacy of a combination of PD-L1 inhibitors (atezolizumab) and VEGF (bevacizumab) and a selective phosphoinositide 3-kinase (PI3K)-gamma inhibitor (IPI-549) in the treatment of metastatic RCC is currently being investigated (NCT03961698). Clinical trials are also underway to evaluate the effectiveness of combined inhibition of BRAF and MEK (MAPK and PI3K-Akt-mTOR signaling pathways) in combination with PD-1/PD-L1 blockade in melanoma [56].

### 3.5. ICs and CXCR4

Overexpression of CXC chemokine receptor 4 (CXCR4) is observed in many types of cancer and is associated with a poor prognosis [57]. CXCR4 is also considered as a promising therapeutic target [58]. A number of clinical trials of the combination of a CXCR4 inhibitor with anti-PD-1/PD-L1 therapy are currently underway in several types of cancer [4].

### 3.6. ICs and EGFR

EGFR expression is associated with the progression of many types of cancer. EGFR is one of the therapeutic targets. It has been shown that EGFR is expressed in 98.4% of cases in ccRCC [59]. A bispecific antibody that inhibits PD-1 and EGFR (anti-PD-1 x anti-EGFR) has been shown to significantly suppress tumor growth and activate antitumor immunity in animal models of various types of cancer [60].

### 3.7. ICs and HER2

Overexpression of the human epidermal growth factor receptor 2 (HER2) occurs in breast and gastric cancer (GC) and is associated with a poor prognosis. Currently, clinical trials are underway to evaluate the effectiveness of a combination of IC and HER2 inhibitors at different stages of the disease [61,62]. A bispecific antibody that simultaneously inhibits PD-1 and HER2 demonstrated significant efficacy in both in vitro and in vivo experiments [63].

## 4. Regulation of IC Genes by miRNAs

Currently, a lot of miRNAs are known to interact with ICs in various types of cancer. Table 1 lists miRNAs that interact with ICs and the type of cancer in which this interaction was shown. More than 50 miRNAs regulate the PD-1/PD-L1 pathway, and about 40 miRNAs regulate B7-H3. For other ICs, fewer miRNA regulators have been described.

In particular, miR-497-5p is a direct inhibitor of PD-L1 in kidney cancer. An inverse correlation was noted between miR-497-5p and PD-L1 expression levels in ccRCC samples. In addition, reduced miR-497-5p expression was associated with shorter survival. In vitro experiments demonstrated that miR-497-5p suppressed tumor cell proliferation and migration, simultaneously stimulating their apoptosis [71].

Other miRNAs also can bind to the 3’-UTR of the *PD-L1* gene and suppress PD-L1 expression. These include miR-570, miR-34a, miR-200, miR-21, and miR-197. Thus, PD-L1 may be the main target for miRNA control of ICs [7].

MiR-138 and miR-28 inhibit PD-1 expression in T-cells. MiR-138 can enhance immune response and slow down tumor progression in mouse [109]. A low level of miR-28 induces T-cell exhaustion and allows tumor cells to evade immune surveillance in a mouse melanoma model [112].

The impact of miRNAs on other ICs may also be important for the development of new approaches in cancer therapy. MiR-448 and miR-153 inhibit IDO1 in CRC. MiR-448 activates CD8+ T-cells by inhibiting function of IDO1 enzyme [115].

MiR-153 inhibits IDO1 expression in CRC cells; however, overexpression of this miRNA does not have a significant effect on tumor cells. However, overexpression of miR-153 has been shown to enhance the effect of chimeric antigen receptor (CAR) T-cell therapy [116].

MiR-128 is a direct inhibitor of Gal-3. Reduced miR-128 expression in CRC was negatively correlated with Gal-3 expression and was associated with poor prognosis. MiR-128 overexpression increased tumor cell sensitivity to chemotherapy in experiments in vitro and in vivo [118].

MiR-498 is an inhibitor of Tim-3. In experiments in AML cell lines, miR-498 significantly suppressed Tim-3 expression, which led to a decrease in proliferation and an increase in cell apoptosis [114].

B7-H3 inhibitors include miR-145, miR-1301-3p, miR-335-5p, miR-28-5p and miR-187. B7-H3 and miR-145 have been shown to be associated with lymph node metastasis, grade, and TNM stage in lung cancer with malignant pleural effusion [124]. MiR-1301-3p, miR-335-5p and miR-28-5p downregulate B7-H3 and are associated with lymph node metastasis and TNM staging in CRC [125]. MiR-187 is also a direct inhibitor of B7-H3. Reduced miR-187 expression has been shown to be associated with TNM stage in kidney cancer. Overexpression of miR-187 resulted in decreased proliferation and migration of tumor cells in vitro and inhibited tumor growth in vivo [130].

The direct target of miR-32 is BTLA. Experiments in OC cell lines showed that miR-32 overexpression led to a decrease in BTLA expression, resulting in a significant suppression of tumor cell proliferation, migration, and invasion [113].

**Figure 1:**
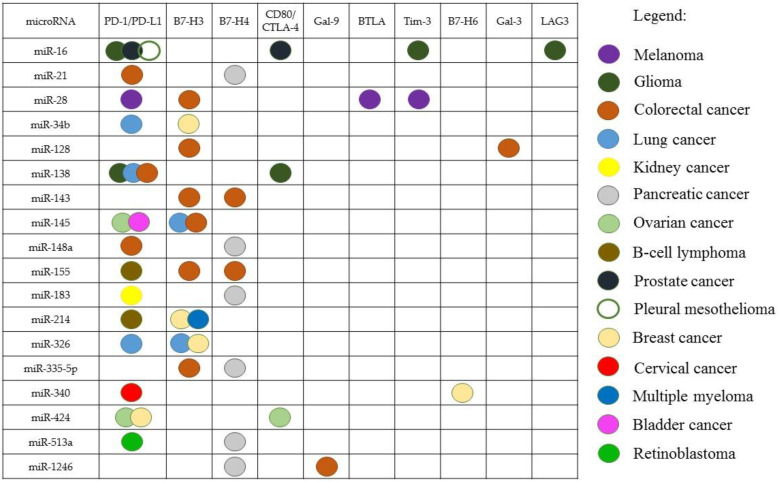
The miRNAs interacting with several IC genes in some types of cancer.

One miRNA can target several ICs in the same type of cancer (Figure 1). For example, miR-28 interacts with PD-1/PD-L1, BTLA, and Tim-3 in melanoma [112]; miR-424 interacts with PD-1/PD-L1 and CD80/CTLA-4 in OC [108]; miR-128 interacts with B7-H3 and Gal-3 in CRC [118,127]; miR-138 interacts with PD-1 and CTLA-4 in glioma [109].

The spectrum of targets of the same miRNA can be different in different types of cancer. In particular, miR-155 interacts with PD-1/PD-L1 in B-cell lymphoma and with B7-H3, B7-H4 in CRC [84,126]; miR-145 acts on PD-1/PD-L1 in OC and bladder cancer [96,97], meanwhile, in lung cancer and CRC, this miRNA acts on B7-H3 [124,126].

It has also been shown that miRNA can interact with the IC gene, in one type of cancer and not in another. An interaction of miR-34 with PD-L1 has been found in lung cancer, but it has not been seen in pleural mesothelioma [81]. On the other hand, the interaction of a certain miRNA with the IC can be observed in several types of cancer: for example, miR-138 inhibits the PD-1/PD-L1 pathway in glioma, CRC, and lung cancer [68,72,109]

**Figure 2:**
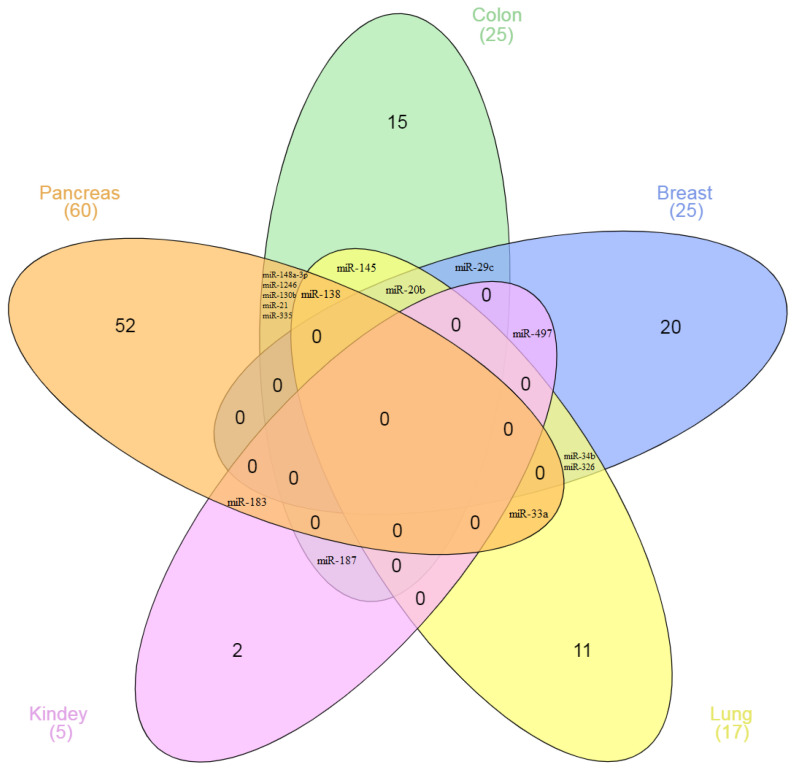
The Venn diagram of the number of miRNAs interacting with IC genes in various types of cancer.

The miRNAs that regulate ICs do so depending on cancer type: there is almost no overlap between these miRNAs in different types of cancer (Figure 2). For example, 60 miRNA regulators of ICs have been identified for PC and 25 for BC. However, among them, no miRNAs have been identified in both PC and BC. The same picture is observed in many other types of cancer. Among 115 miRNAs shown in the diagram, only 15 miRNAs (13%) interact with the IC genes in two or more types of cancer. 

The confidence interval (CI) for the obtained ratio (15/115) was determined. The 95% CI was from 7.3% to 21.5%. The diagram (Figure 2) shows miRNAs in five types of cancer, which are distributed with the specified characteristics. In total, we have considered 207 miRNAs in the article, 30 of which regulate IC in different types of cancer (Table 1). The analysis of the data demonstrates the only 14.5% (95% CI: 9.8–20.1%) of the studied miRNAs regulate the expression of specific IC in more than one type of cancer. That is, when the number of analyzed miRNAs increased by almost two times, the share of overlapping miRNA and confidence intervals practically did not change.

For the first time, on a large sample of miRNAs, we showed the tumor specificity of their action on the IC genes. Similar results were obtained both in the study of five types of cancer and in the study of more of them. These calculations show that the number of miRNAs studied is sufficient for reliable statistical evaluation. Similar results are also observed in separate experiments. Thus, in the work of Qian et al., 62 miRNAs associated with B7-H4 in pancreatic cancer were identified using the miRCURY LNA™ microchip. Of these miRNAs, only 8 (13%) regulate ICs in other cancers [143]. In lung cancer, it was shown that miR-34 directly interacts with the 3’-UTR of PD-L1; miR-34 overexpression suppressed PD-L1 protein expression [87]. At the same time, in pleural mesothelioma, Kao et al. did not observe the relationship between the expression of miR-34 and PD-L1 [81]. Thus, our conclusion about the tumor-specific interaction of miRNA with ICs is consistent with the results of studies by other authors. Essentially, miRNAs can be differentially expressed depending on cancer type. Korotaeva et al. found that miRNAs are specifically expressed in certain types of neuroendocrine tumors [145].

## 5. miRNAs as Regulators of ICs and Targeted Therapy Genes

In addition to ICs, miRNAs can also affect other genes (such as mediators of angiogenesis, hypoxia, etc.) and thus regulate different signaling pathways (Table 2). In other words, miRNAs function as part of a complex signaling network [146].

In particular, in CRC, a number of miRNAs may affect both ICs and other genes that play a key role in cancer development. For example, miR-145 inhibits its target B7-H3, miR-143 is a direct inhibitor of B7-H3 and B7-H4. Also, these miRNAs suppress the angiogenesis regulator VEGF, HIF1 and the IRS-1/IGF-IR signaling pathway, which is a potent inhibitor of apoptosis and cell differentiation [126,157,158,159].

MiR-143 indirectly interacts with miR-155. The miR-155 targets C/EBPβ, a transcriptional activator for miR-143. MiR-155, by inhibiting miR-143, has been shown to increase the expression of B7-H3 and B7-H4 [126]. Also, miR-155 is described as a direct inhibitor of HIF-1α, and thus, being a regulator of various signaling pathways, miR-155 functions as an oncomiR and a tumor suppressor [156].

MiR-148a, in addition to PD-L1, inhibits the human epidermal growth factor receptor 3 (HER3), Wnt/β-catenin pathway ligand—WNT10b, apoptosis regulator—BCL2, as well as VEGF and HIF1 in CRC; that is, it acts as a tumor suppressor [73,164,165,166,167]. 

In addition, miR-148a has been shown to inhibit the calnexin (CANX)/MHC-I signaling pathway in CRC, by downregulating its direct target, CANX. An increased level of CANX expression in the tumor positively correlates with the overall survival of patients with CRC. Thus, miR-148a functions as a tumor promoter in this case [168].

In CRC miR-1246 inhibits Gal-9 and CCNG2, which is a tumor suppressor; decreased expression of CCNG2 occurs in many types of cancer and correlates with lymph node metastasis, clinical stage, and poor prognosis [121,163].

One miRNA can be involved in several signaling pathways that mediate different, sometimes opposite, cell functions. This can lead to the development of adverse events of miRNA-based therapy. Thus, when considering miRNAs as therapeutic targets, the features of their action in a particular type of cancer should be taken into account.

## 6. Prospects for miRNA-Based Therapy

Currently, a number of therapeutic approaches involving miRNAs are being developed: combinations of miRNAs with other agents [173,174,175,176,177], as well as combinations of two miRNAs [178,179,180] are being investigated.

MiRNAs from the miR-200 family are inhibitors of PD-L1 [90], HIF-1α, and the VEGF pathway [181,182,183]. According to Nguyen et al., nanoparticle therapy containing miR-200c as a PD-L1 inhibitor with a BRAF inhibitor demonstrated significant efficacy in a mouse model of CRC [177].

MiR-15/16 cluster miRNAs have been shown to interact with several genes. In prostate cancer, a negative correlation was found between the expression level of miR-195 and miR-16 and PD-L1, PD-1, CD80, and CTLA-4 [86]. In pleural mesothelioma, miR-15/16 inhibited PD-L1, Bcl-2, and CCND1. Injection of miR-16 mimetics led to a decrease in Bcl-2 and CCND1 expression levels and also inhibited tumor growth in animal models of malignant pleural mesothelioma [81,147]. The efficacy of miR-16 mimetic was evaluated in phase I clinical trials in patients with malignant pleural mesothelioma. The trial results were described as promising and suggest further drug research [184,185].

Experiments on T-cells have shown that direct targets for miR-155 are CTLA-4 and BTLA [186,187]. Administration of antibodies against CTLA-4, PD-1, and PD-L1 activated the antitumor immune response in miR-155 knockout mice [188]. MiR-155 also indirectly inhibited CXCR4 in glioma [156,189]. In chronic lymphocytic leukemia, diffuse large B-cell lymphoma, and liposarcoma, miR-155 acts as an oncomiR by inhibiting its direct targets, the casein kinase CSNK1G2 and the casein kinase 1α (CK1α) isoforms [190,191]. Also, miR-155 upregulates PD-L1 expression [84] and regulates a number of signaling pathways (including JAK/STAT, MAPK/ERK, and PI3K/AKT) in lymphoma [192]. The miR-155 inhibitor has performed well in the clinical trials NCT02580552, NCT03837457 and NCT03713320 in patients with hematologic malignancies in which miR-155 is overexpressed, such as cutaneous T-cell lymphoma, mycosis fungoides, chronic lymphocytic leukemia, diffuse large B-cell lymphoma, ABC subtype, adult T-cell leukemia/lymphoma [193].

MiR-138 acts as a tumor suppressor in many types of cancer and inhibits many target genes. Overexpression of miR-138 may increase the sensitivity of tumor tissue sensitivity to chemotherapy [194]. In lung cancer, miR-138 has been shown to significantly inhibit tumor cell proliferation in vitro by acting on its target, the PD-L1/PD-1 pathway. In experiments on NSCLC xenografts, miR-138 not only inhibited tumor growth, which led to a decrease in its size, but also regulated the tumor microenvironment [68]. In CRC, transfection with miR-138-5p mimetics also led to a decrease in tumor cell proliferation. In experiments on xenografts with the administration of miR-138 tumor size was decreased [72]. In glioma models, miR-138 inhibited CTLA-4 and PD-1, leading to significant regression of subcutaneous tumors. Moreover, tumor regression continued even after treatment was stopped. The administration of miR-138 was also effective in animals with intracerebral tumor localization: the average lifespan of mice treated with miR-138 was 33.5 days, and in the control group it was 23.5 days [109].

MiR-424 is a direct inhibitor of PD-L1 and CD80. It was shown that miR-424 expression in OC tumors negatively correlated with the PD-L1 and CD80 expression level. Also, miR-424 overexpression was correlated with progression-free survival. In experiments in animal models of OC, restoration of miR-424 expression increased tumor sensitivity to chemotherapy. Administration of miR-424 led to tumor regression and decreased tumor cell chemoresistance due to the activation of the T-cell immune response [108].

MiR-142-5p is a direct inhibitor of PD-L1. In vitro experiments revealed that miR-142-5p overexpression did not affect the tumor cell proliferation. However, miR-142-5p has been shown to inhibit the growth of PC and enhance antitumor immunity in vivo [69].

It has been shown that miR-34a inhibits PD-L1 and reduces the proliferation and migration of BC cells [195]. Overall, more than 30 target genes have been described for miR-34a involved in various signaling pathways in cancer. MiR-34a liposomal mimic, MRX34, is the first-in-human miRNA-based drug that has been evaluated in clinical trials in patients with various solid tumors [196]. This trial was terminated due to serious immune-mediated adverse events that may indicate the effect of this miRNA on several immune system regulatory genes. Despite this, miR-34a continues to be considered as a therapeutic target in cancer, as it has significant tumor suppressor potential. It was shown that the induced co-expression of miR-34a with other miRNAs led to a pronounced and stable therapeutic effect in models of various types of cancer, such as CRC, NSCLC, melanoma, etc. [197]. Orellana et al. identified five miRNAs that, in combination with miR-34a, most effectively inhibited tumor cell proliferation [198]. The combination of miR-34a-mimic and antisense-miR-10b is also being studied in BC models [180]. Meng et al. showed that the synthesized miR-34a analog NS-MX3 simultaneously decreased the expression of B7-H3 and PD-L1 and demonstrated superior antitumor activity in CRC models in vitro and in vivo [199].

Thus, the results of in vitro and in vivo experiments on the study of miRNAs as immunotherapeutic agents look encouraging.

In order to use miRNA-based drugs in clinical practice, these drugs must meet the requirements for efficacy and safety. Techniques that provide more specific binding to a target can increase the effectiveness of miRNA-based therapy, as well as reduce the dose of the administered drug, thereby reducing its side effects.

## 7. Conclusions

We have reviewed more than 200 miRNAs that regulate ICs in tumors of various types. The results of accumulated data analysis for the first time demonstrate a significant relationship between the action of miRNAs on ICs genes and the type of tumor—only about 14% (95% CI: 9.8–20.1%) of the studied miRNAs regulate the expression of specific IC in more than one type of cancer. That is, there is tumor specificity in the miRNA action on ICs. 

An important feature of miRNA is its ability to affect the expression of several genes simultaneously. The data described here evidenced that some miRNAs can simultaneously regulate more than one IC gene. In addition, there are miRNAs that can affect both the IC gene and some targeted therapy genes. These results indicate the possibility of using miRNAs in the future as an alternative to combined treatment regimens that use inhibition of both two ICs simultaneously and inhibition of ICs together with targeted therapy genes.

Currently, there are numerous studies underway to identify miRNAs that are the most promising as immunotherapy agents. In vivo experiments have repeatedly shown that miRNA-based therapy leads to significant tumor regression.

Although miRNA has not yet entered the arsenal of antitumor agents used in practice, some results are encouraging. Thus, the miR-155 inhibitor has performed well in clinical trials. The study of miR-138 is promising. Ongoing research on miR-34a may also lead to a positive result. Thus, there is the prospect of using miRNA as a therapeutic agent in cancer immunotherapy regimens.

At the same time, the ability of miRNAs to inhibit several genes can lead to adverse events. To overcome this, it is important to expand data of the spectrum of miRNA targets in a particular type of cancer. Additional studies of the miRNA–genes interaction features and the search for an optimal miRNA mimic structure are necessary, thus allowing an increase in the efficiency and selectivity of interaction with the mRNA of target genes. It can increase the effectiveness of therapy, as well as reduce the dose of the drug, thereby reducing its side effects.

## Figures and Tables

**Table 1:** The miRNAs interacting with IC genes in different types of cancer.

Immune Checkpoint	microRNA	Cancer	Reference
PD-1	miR-374b, miR-4717	Liver cancer	[64,65]
PD-1/PD-L1	miR-183	RCC	[66]
	miR-138-5p, miR-200b, miR-429, miR-508	Lung cancer	[67,68]
PD-L1	miR-142-5p	PC, OC	[69,70]
	miR-497-5p	ccRCC	[71]
	miR-20-b, miR-21, miR-130b, miR-138-5p, miR-148a-3p, miR-191-5p	CRC	[11,72,73,74]
	miR-195, miR-424-5p, miR-497, miR-873, miR-3609	BC	[75,76,77,78]
	miR-17-5p, miR-146a	Melanoma	[79,80]
	miR-15a, miR-15b, miR-16, miR-193a-3p, miR-320a	Pleural Mesothelioma	[81,82]
	miR-155, miR-195, miR-214	B-cell lymphoma	[83,84,85]
	miR-16, miR-195	Prostate cancer	[86]
	miR-34a, miR-34b, miR-34c, miR-140, miR-200, miR-200a-3p, miR-3127-5p	Lung cancer	[87,88,89,90,91]
	miR-34a	AML	[92]
	miR-23a-3p, miR-570	Liver cancer	[93,94]
	miR-375	HNSCC	[95]
	miR-145	OC, bladder cancer	[96,97]
	miR-513a-5p	Retinoblastoma	[98]
	miR-105-5p, miR-152, miR-200b, miR-200c, miR-570	GC	[99,100,101,102,103]
	miR-18a, miR-140, miR-142, miR-340, miR-383	Cervical cancer	[104]
	miR-217	Laryngeal cancer	[105]
	miR-20b-5p	Models of lung and BC	[106]
	miR-194-5p	PC	[107]
PD-L1+B7-H3	miR-326	Lung cancer	[8]
PD-1, CTLA-4	miR-424	OC	[108]
	miR-138-5p	Glioma	[109]
CD80/CTLA-4	miR-424	CRC	[110]
PD-1, PD-L1, CTLA-4	miR-33a	Lung cancer	[111]
PD-1, BTLA, Tim-3	miR-28	Melanoma mouse model	[112]
BTLA	miR-32	OC	[113]
Tim-3	miR-498	AML	[114]
IDO1	miR-153, miR-448	CRC	[115,116]
Gal-3	miR-424-3p	OC	[117]
	miR-128	CRC	[118]
Gal-9	miR-22	Liver cancer	[119]
	miR-15b-5p, miR-455-5p, miR-1237, miR-1246	CRC	[120,121]
ICOS (B7-H2)/ICOSL	miR-24	GC	[122]
B7-H3	miR-29 (a, b and c)	Neuroblastoma, sarcoma, brain tumors	[123]
	miR-145	Lung cancer	[124]
	miR-28-5p, miR-29a, miR-128, miR-145, miR-155/miR-143, miR-187, miR-192, miR-335-5p, miR-378, miR-1301-3p	CRC	[125,126,127,128,129]
	miR-187	ccRCC	[130]
	miR-29c	Melanoma, CRC	[131,132]
	miR-29c, miR-34b, miR-124a, miR-125b-2, miR-214, miR-297, miR-326, miR-363, miR-380-5p, miR-506, miR-555, miR-567, miR-593, miR-601, miR-665, miR-708, miR-885-3p, miR-940	BC	[133]
	miR-539	Glioma	[134]
	miR-124	Osteosarcoma	[135]
	miR-506	Mantle cell lymphoma	[136]
	miR-214	Multiple myeloma	[137]
	miR-29, miR-1253	Medulloblastoma	[138,139]
	miR-199a	Cervical cancer	[140]
B7-H5 (VISTA, BTNL2)	miR-125a-5p	GC	[141]
B7-H4 (VTCN1)	miR-155/miR-143, miR-1207	CRC	[126,142]
	miR-7–5p, hsa-let-7c, hsa-let-7f-5p, miR-17–3p, miR-21–3p, miR-21–5p, miR-24–1-5p, miR-27b-3p, miR-31–3p, miR-31–5p, miR-33a-5p, miR-33b-5p, miR-122–3p, miR-130b-3p, miR-138–1-3p, miR-148a-3p, miR-149–3p, miR-183–3p, miR-186–5p, miR-196a-5p, hsa-miR-204–3p, miR-299–5p, miR-302a-3p, miR-302e, miR-335–3p, miR-335–5p, miR-361–5p, miR-374c-5p, miR-483–3p, miR-513a-5p, miR-519e-3p, miR-520d-5p, miR-525–5p, miR-615–3p, miR-642a-5p, miR-744–5p, miR-937, miR-1246, miRPlus-G1246–3p, miR-1260a, miR-1265, miR-1284, miR-1290, miR-1973, miR-2115–3p, miR-2116–5p, miR-3178, miR-3202, miR-3646, miR-3651, miR-3676–3p, miR-3685, miR-3686, miR-4258, miR-4279, miR-4284, miR-4288, miR-4290, miR-4306, miR-4324	PC	[143]
B7-H6 (NCR3LG1)	miR-93, miR-195, miR-340	BC	[76]
B7-H7 (HHLA2)	miR-3116, miR-6870-5p	ccRCC	[144]

Footnotes: RCC—renal cell cancer; PC—pancreatic cancer; OC—ovarian cancer; CRC—colorectal cancer; BC—breast cancer; AML—acute myeloid leukemia; HNSCC—head and neck squamous cell cancer; GC—gastric cancer.

**Table 2:** MiRNAs regulating immune checkpoints as well as other cancer relevant genes.

microRNA	Immune Checkpoints	Other Targets	Cancer	Reference
miR-16	PD-1/PD-L1	BCL2, CCND1	Pleural mesothelioma	[81,147]
miR-138	PD-1/PD-L1, CD80/CTLA-4	CD44, SOX13	Glioma	[109,148,149]
	PD-1/PD-L1	SIRT1	CRC	[72,150]
miR-34a	PD-1/PD-L1	EGFR, BCL-2, Met	Lung cancer	[87,151,152]
miR-326	PD-1/PD-L1, B7-H3	CCND1, ADAM17		[8,153,154]
miR-340	B7-H6	MET	BC	[76,155]
miR-155	B7-H3, B7-H4	HIF-1	CRC	[126,156]
miR-143 and miR-145	B7-H3, B7-H4	VEGF/VEGFR, HIF-1, IRS-1/IGF-IR		[126,157,158,159]
miR-335	B7-H3	ZEB2		[125,160]
miR-128	B7-H3, Gal-3	SIRT1		[118,127,161]
miR-28	B7-H3	CCND1		[125,162]
miR-1246	Gal-9	CCNG2		[121,163]
miR-21	PD-1/PD-L1	PTEN		[11]
miR-148a	PD-1/PD-L1	HER3, WNT10b, VEGF/VEGFR, HIF-1, BCL-2, CANX		[73,164,165,166,167,168]
miR-424	PD-1/PD-L1	PTEN, IRS-1/IGF-IR, BCL-2	BC	[78,169]
miR-214	B7-H3	PTEN		[133,170]
miR-183	B7-H4	PDCD4	PC	[143,171]
miR-142	PD-1/PD-L1	BIRC3, BCL2, BCL2L2, MCL1, XIAP	OC	[70,172]

## Data Availability

The cited works can be found on the PubMed, PMC, Omicsonline, and Embase databases.
